# ‘*Healthier options tend to get lost in the noise of online’* – Australian shoppers’ experiences with online grocery platforms

**DOI:** 10.1017/S1368980024001046

**Published:** 2024-05-14

**Authors:** Rebecca Bennett, Christine Driessen, Christina Zorbas, Gary Sacks, Adyya Gupta, Adrian Cameron, Clara Gomez-Donoso, Anna Peeters, Kathryn Backholer

**Affiliations:** Global Centre for Preventive Health and Nutrition, Institute of Health Transformation, Deakin University, Geelong, Australia

**Keywords:** Australia, Food environment, Online grocery, Digital food retail environment, Online shopping

## Abstract

**Objective:**

We aimed to understand what influences parents’ purchasing behaviours when shopping for groceries online and potential ways to improve the healthiness of online grocery platforms.

**Design:**

We conducted semi-structured interviews, guided by the Marketing Mix framework. Reflexive thematic analysis was used to analyse data.

**Setting:**

Online interviews were conducted with primary grocery shoppers.

**Participants:**

Parents (*n* 14) or caregivers (*n* 2) using online grocery platforms at least every 2 weeks.

**Results:**

Most participants perceived purchasing healthy food when shopping for groceries online to be more challenging compared to in physical stores. They expressed concerns about the prominence of online marketing for unhealthy food. Participants from lower socio-economic backgrounds often depended on online supermarket catalogues to find price promotions, but healthy options at discounted prices were limited. Across socio-economic groups, fresh items like meat and fruit were preferred to be purchased instore due to concerns about online food quality.

Participants believed online grocery platforms should make healthy foods more affordable and supported regulations on supermarket retailers to promote healthy options and limit unhealthy food promotion online.

**Conclusions:**

Participants had varied experiences with online grocery shopping, with both positive and negative aspects. Efforts to improve population diets need to include mechanisms to create health-enabling online grocery retail platforms. Government interventions to restrict marketing of unhealthy foods and promote marketing of healthy options on these platforms warrant investigation.

Unhealthy diets and obesity are major public health problems globally^(1)^, with 39 % of the world’s adult population living with either overweight or obesity in 2020^(2)^. In high-income countries, obesity disproportionately affects those who experience socio-economic disadvantage^(3)^. For example, in 2017–18 in Australia, the prevalence of obesity was 38 % among those living in the most disadvantaged areas (lowest area-level socio-economic status), compared to 24 % in those living in the least disadvantaged areas (highest area-level socio-economic status)^(4)^. Unhealthy diets, characterised by a high intake of highly processed, energy-dense foods and beverages and a low intake of fruits, vegetables and legumes, are also socio-economically patterned, with lower area-level socio-economic status associated with poorer diet quality, and drive inequalities in overweight and obesity^(5)^. The shopping and eating habits of households with children are of particular interest for public health, given the influence of childhood diets and body weight on the risk of adult obesity and continued unhealthy eating habits^(6)^.

In Australia, supermarkets make up 63 % of food-related expenditure^(7)^, with two major retailers having 65 % market share^(8)^. They are extremely similar in their offerings, layout and promotional techniques, both instore and online. There has been considerable research to understand how supermarket environments shape instore shopper behaviour^(9,10)^ with retailers using ‘cues’ and ‘purchase triggers’ to encourage shoppers towards certain products^(10)^. Store layout, prominent promotional displays, price discounting and signage (among other strategies) are all successfully used to influence purchasing^(10)^. Australian supermarkets have been found to use price promotions extensively, with a greater prevalence and magnitude of price promotions on unhealthy foods compared to healthy options^(11)^. However, increasingly households are using online grocery platforms to order foods and beverages, particularly since the advent of the COVID-19 pandemic^(12)^. In Australia, recent total online spending for food (including online grocery) has more than doubled – rising from AUD$454 million in May 2019 to AUD$1112 million in May 2023^(13)^. In the financial year 2023, online sales for the two major Australian supermarkets totalled over AU$9·4 billion^(14,15)^. Previous studies have shown that households with children are more likely to use online grocery shopping than household without children^(16)^. Online grocery environments can influence shopping behaviour in different ways to physical stores, for example, through algorithmic personalisation of search results, web design and page layouts^(17)^. Limited evidence exists describing how online grocery platforms potentially influence purchasing behaviour and through what mechanisms, with none that we are aware of in the Australian context^(18)^.

We aimed to:

1. Understand online grocery shopping behaviours, and participant perceptions of what influences these purchasing behaviours, of school-aged children’s parents and carers when shopping for groceries online.
2. Understand the perceptions of parents and carers regarding how to improve the healthiness of online grocery retail platforms.

## Methods

We used a qualitative descriptive study design to gain an exploratory understanding of Australian shoppers’ perceptions of the online grocery retail environment. Semi-structured in-depth interviews were conducted with grocery shoppers from households with school-aged children.

This study follows reporting guidelines for qualitative interviews, as described by the COREQ checklist^(19)^.

### Research team and reflexivity

Interviews for this study were conducted solely by the first author (RB), a female PhD student with 5 years of experience working in health research. RB is a parent with two young children, one of whom is school-aged, and has not previously experienced living on a low income. Additionally, RB has used online grocery platforms for the previous 5 years and is familiar with both major Australian supermarkets’ websites and shopping apps. The research team has extensive expertise in healthy food retail research in the Australian and international context, including with online platforms. Prior to these interviews, no participants had any previous contact with the research team.

### Study design

#### Theoretical framework

The Marketing Mix framework^(20)^ was used as the theoretical basis for the formulation of the interview guide. This framework has been used previously to analyse the influences of supermarkets on purchasing behaviour^(21)^, with some limited application to the online retail context^(22)^. The Marketing Mix theory is a fundamental cornerstone of corporate marketing strategies, comprising four elements – product, price, place and promotion – for engaging customers and encouraging purchases^(23)^.

The semi-structured interview guide was developed through collaboration between authors, discussing key differences between the experience of shopping online for groceries compared to instore.

#### Participant selection

Participants were recruited using a paid recruitment company, located in Melbourne, Australia. Initial contact with potential participants was facilitated through the recruitment company, by sending an email to their database of users. If potential participants were interested, they were directed to the screening questionnaire and a copy of the plain language statement and consent forms. Once the screening questionnaire was completed, the recruitment company arranged a suitable interview time. Participants were informed that interviews were being conducted as part of research examining the digital food retail environment and its impact on diet and health.

#### Inclusion criteria

A screening questionnaire was completed to ensure that participants met the inclusion criteria, which included: (i) a parent or carer of a school-aged child (primary or secondary, typically aged between 5 and 18 years), (ii) using online grocery platforms at least once every 2 weeks and (iii) fluent in English. Demographic information was also collected once a participant passed the screening questionnaire, including age, household income, postcode (this was then converted by the researchers into a Socio-Economic Indexes for Areas (SEIFA) Index of Relative Socio-economic Disadvantage (IRSD) quintile^(24)^), sex, highest level of education, household size and whether they belonged to a single- or double-income household

As a key aim of our study was to understand differences in perspectives of online grocery shopping across socio-economic groups, recruitment was monitored to achieve a quota of approximately half of the sample being classified as ‘low income’ through regular contact with the recruitment company. Low income was defined as having a total household income of less than $1400 per fortnight, which is approximately equivalent to the 2021 median Australian fortnightly household income after tax^(25)^ and is approximately equal to the first two quintiles of the Australian equivalised disposable household income^(26)^. Participants whose household income was greater than $1400 per fortnight were classified as ‘mid-high income’.

Additionally, a sampling quota was used to achieve an approximately even split of women and men (independent of socio-economic position), as men have traditionally been a more difficult group to recruit for research relating to grocery shopping^(27)^. No quotas were set regarding household location or educational attainment of participants.

Data saturation was determined as the point at which no new concepts or ideas were elicited across the interviews and when there was no longer a need for new codes to be created during data analysis^(28)^.

#### Data collection

The interview guide was pilot-tested by RB with another author (CD) (who met the inclusion criteria) before participant interviews began. An interview guide of core questions is provided in Appendix A.

Thirty- to sixty-minute interviews were conducted remotely, using the Zoom software platform in November and December 2022. An audio-only recording of the interview was created using the Zoom platform for transcription purposes. Following transcription, participants were emailed a copy of their interview transcript for comment and/or approval before it was included for analysis.

#### Data analysis and reporting

Data were managed and analysed using QSR NVivo 20 software (IQR International Software). Following the conclusion of the interview process, reflexive thematic analysis was undertaken using the six-step method outlined by Braun and Clarke^(29)^.

One researcher (RB) familiarised herself with the data by rereading the interview transcripts and making initial notes. Collaborative coding of the first interview was undertaken by two authors (RB and CD) to increase the richness and depth of understanding, with all remaining interviews coded by one researcher (RB). Following this, initial coding of the remaining transcripts was undertaken using three components of the Marketing Mix framework (price, promotion and place). This framework guided iterative and inductive assignment of codes with a view to identify common themes across the interviews. After initial coding was complete, early themes were constructed by grouping together similar codes. Perspectives based on participant socio-economic position were coded. Final themes were derived through discussion with three authors (RB, CZ and KB). Quotations from participants were used to illustrate themes and findings.

## Results

### Overview and participant characteristics

Sixteen interviews were conducted, with an average length of 36 min.

Interviews were primarily conducted with mothers and fathers of school-aged children, with two grandparent carers also participating. The mean age of parent participants was 38 years, and the mean age of grandparent participants was 57 years. Most participants had a bachelor’s degree or higher, lived in a three-person household and lived in a single-income household. Two participants reported health conditions that made shopping instore for groceries difficult. Despite aiming for half of all participants to be from low-income households, only 38 % of participants were recruited from this group due to interview cancellations. Similarly, we were only able to achieve a proportion of 37 % male participants.

A full summary of participant characteristics is reported in Table 1:

**Table 1:** Summary of demographic characteristics of participants (*n* 16)

Participant characteristics		
Age (mean, sd)	Parent (*n* 14)	38, 3·7
	Grandparent (*n* 2)	57, 3·5
Female (%)		63 (*n* 10)
Education level (%):		
High school		6 (*n* 1)
TAFE or technical certificate		38 (*n* 6)
Bachelor’s degree or higher		56 (*n* 9)
Postcode SEIFA IRSD* quintile (%)		
1 (most disadvantaged)		0
2		6 (*n* 1)
3		38 (*n* 6)
4		25 (*n* 4)
5 (least disadvantaged)		31 (*n* 5)
Disposable income below $1400 per fortnight (%)		38 (*n* 6)
Household size (%):		
3		63 (*n* 10)
4		37 (*n* 6)
Single-income household (%)		56 (*n* 9)

* SEIFA, Socio-Economic Indexes for Areas; IRSD, Index of Relative Socio-economic Disadvantage.

Overall, twenty-five major codes were used to construct five themes from the data. These are reported below according to each of the aims, with differences by socio-economic position highlighted throughout the text. Data saturation occurred across the interviews, irrespective of income level. A full list of major codes is available in Appendix B. A summary of results can be found in Table 2:

**Table 2:** A summary of themes, including key quotes from participants

Theme	Description	Participant quotes
Online promotions are useful, but unhealthy food specials dominate	Participants used online price promotions to save money but felt that there was imbalance in the healthiness of items promoted.	*[On special are], all your foods that aren’t healthy. I’m sorry, but it’s true. You can buy chocolate and ice cream cheap, cheaper than if you buy cauliflower, or anything like that.* **(55-year-old female, low income)** *The most prominent things on promotion are chocolate, chips, snack chips. And yeah microwave meals. So yeah, it’s not very healthy.* **(40-year-old female, mid-high income)** *I go through the catalogues because I get the catalogue sent to me online. Of course I’ll go through the catalogues and see what’s on special*. **(40-year-old female, mid-high income)** *I’ll go into the catalogue section and scroll through and see what’s available and what’s on special at the time… And yeah, choose which foods I’m going to be looking at cooking for that next week.* **(37-year-old female, low income)**
Online grocery platforms are helpful for budget-conscious shoppers	Participants felt that using an online platform to grocery shop (instead of shopping instore) allowed them to plan purchases more carefully and stay to their budget.	*Price pays a lot into what people buy… I think we’re all aware of what’s healthy and what’s not healthy. But if you’re working to a budget. We’ve only got so much money*. **(55-year-old female, low income)** *Put the costs down a bit so we can at least eat a bit healthier*. **(60-year-old female, low income)** *Because you just keep dropping it into your [online basket]and you can go in there anytime you like, and it tells you how much it is. Whereas sometimes you get a bit of a shock when you buy a whole heap of stuff [instore] and you get to check out and think ‘Oh my God, it’s really that much?’* **(50-year-old male, mid-high income)** *Because it calculates [the total] for you, whereas in the store I don’t really have the time to walk around with the calculator… And then when you get to the register it’s like ‘Ohh OK, I’ve spent a bit too much today’*. **(40-year-old female, mid-high income)**
There are pros and cons with shopping instore and online	Participants described how there are benefits to shopping online (convenience, do not need to shop with children) but also negatives (dissatisfaction with perishable item quality).	*You’ve got time to sit down in your comfort and gloss over the catalogues and what’s available and all the specials that come in and then put them all into your cart. So it’s just a lot more time that you can spend on doing your grocery lists then say, you know, if you were just in the supermarket you’re running around with the child and…. I mean you can carefully plan lot more things*. **(55-year-old female, low income)** *Walking in the shop you get to see the produce. If it does look good, seasonal fruit… I’ll buy if I see them and they look good. The alternative, like I said, you can’t do that online shopping.* **(40-year-old male, mid-high income)** *I know that I’m less likely to be shopping with my eyes [online]*. **(38-year-old female, low income)**
Scepticism of supermarket-led actions	Participants were distrustful of supermarkets and suggested a range of ways to improve the healthiness of online grocery purchasing.	*At the same time, I understand supermarkets have to make money. So it makes sense for them to advertise [both healthy and unhealthy food].* **(40-year-old male, low income)** *Everyone knows, wholemeal [is] more healthy… To me, it’s like they just want to get that little bit more money because they’re dearer. Why don’t they have [the healthy bread] on special or, $3·40 or $3·10 instead of $3·90? It’s yeah, a bit of a price gouge.* **(60-year-old female, low income)** *I also think the government has a… there’s a responsibility to the people and almost a duty of care, in a way.* **(32-year-old male, mid-high income)**
A role for government-led actions	Participants generally agreed that there was a place for government regulation of online supermarkets, though they felt this could be difficult due to resistance from supermarkets.	*[Companies] that produce those types of [unhealthy] items, I would suggest to you that because they make so much money out of those areas that they’ll never… They’re not gonna do it willingly. I’ll just put it that way to you*. **(50-year-old male, mid-high income)** *I would also think that the government would benefit from healthier food purchases and healthier lifestyles. Because then that would eventually impact on the cost of the health system.* **(60-year-old male, low income)** *It should be a maybe a policy… I think a health promotion policy to say that when they stop advertising cigarettes, for example, you know or they change that now the packaging has the bad health effects that has on the human body…. They should do similar things for junk food*. **(43-year-old female, mid-high income)**

### Perceived influences of online grocery shopping on purchasing behaviours

#### Theme 1: online promotions are useful, but unhealthy food specials dominate

Participants felt that the promotion of unhealthy food and beverages was more prominent than the promotion of healthy food and beverages when shopping online. They discussed how they had intentions to eat a healthy diet but found it difficult when so much unhealthy food was discounted and advertised online. Participants also discussed how the combination of unhealthy food marketing in the online and physical food retail environment made them feel as though they were ‘saturated’ with messages encouraging the consumption of unhealthy foods. There was some perception that this marketing of unhealthy foods on online grocery platforms influences purchases:

*Everything that’s advertised [online], it’s junk food, and easily accessible food. And the more it’s advertised, the more people are inclined to want to purchase and to want to try, or to want to have it.* **(37-year-old female, low income)**

Specifically, participants felt supermarkets’ websites were ‘prompting’ them to look at the price promotions, by featuring a large website banner on the homepage that advertised discounted products and by having the ‘specials’ tab at the top of each page of the website. They also discussed the personalised list of price promotions that they receive either through the supermarket website or via email, which they mostly perceived as useful to help them save money. There were mixed opinions as to whether food and beverage price promotions were more visible online or instore.

Participants discussed the usefulness of the online version of the supermarket catalogue when completing their online shop, specifically to try to find price-promoted food and beverages. Participants used this to directly add promoted products to their cart, by clicking links embedded in the online catalogue. This feature was emphasised by participants on a low income, who indicated that reading the online catalogue was usually one of the first things they did when shopping online. Similarly, using the ‘specials’ or ‘online only’ tab on the website to find price promotions was a common way to begin planning an online shop. However, participants were generally dissatisfied with the types of food and drinks on price promotion online, commenting that it was mostly unhealthy, processed food and that not enough healthier options were discounted. Price promotions for fruits and vegetables were reported as being uncommon, which was frustrating to participants. According to one participant:

*I’ve never seen any healthy things on special or discount or being promoted [online].* **(38-year-old female, mid-high income)**

Another promotional technique that participants viewed as useful was the online recipes, which were reported to provide a useful way to encourage shoppers to try different ways of preparing fruits and vegetables, especially when ingredients could be conveniently added directly to their online shopping cart. Some commented that they had used the recipes before and found them useful for healthy meal ideas that can be prepared on a budget. Participants with lower income reported that they found the online recipes particularly useful.

Personalised checkout prompts used to suggest recently purchased items that were not selected in the current shop (e.g. the ‘Have you forgotten?’ prompt) were also noticed by most participants. Participants discussed, again, that these were generally promoting unhealthy food and beverages. Other forms of promotions (e.g. items promoted through search optimisation) were generally either deliberately ignored or not noticed by most participants.

#### Theme 2: online grocery platforms are helpful for budget-conscious shoppers

The price of a product was described as a key motivator when deciding what to buy online. Participants perceived the relatively stable and lower price of unhealthy processed foods compared to the highly variable and higher costs of fresh fruits and vegetables as challenging, particularly for participants with lower incomes who were budgeting for groceries.

Nevertheless, most participants perceived that online grocery shopping enabled them to plan more carefully what they wanted to buy compared to shopping instore, thereby reducing impulse buying and helping to manage their spending. Sorting search results by unit price (price per kilogram, litre, etc) allowed participants to select the cheapest item, which was reported to be particularly useful when buying staple products like milk. Participants also reported choosing cheaper fruit and vegetables, often less expensive ‘imperfect’ products, to further save money. On the other hand, participants with lower income commented that they were unable to purchase cheaper ‘reduced to clear’ items that were close to expiry when shopping online – a strategy which they used to save money when shopping instore.

Other strategies described by participants to keep to their budget included planned shops through the use of lists (physical or using a list-making app). Participants also often discussed how ‘bought before’ lists (generated by the online supermarkets) and the online grocery cart feature allowed them to purchase relatively cheaper products and keep track of their spending as they added each item to their cart. Participants described how this was in contrast to shopping instore when it can be an unpleasant surprise to find out the total cost of their purchases at the checkout. With online shopping, items can be added or removed to achieve a total price that you are comfortable to pay:

*In the shop when you get to the checkout it can get a bit awkward [if the basket exceeds your budget] but on the online it doesn’t*. **(40-year-old male, mid-high income)**

In contrast, some participants reported spending more money online compared to instore which was generally because they did not feel as rushed when shopping online and had more time to ensure that they had everything they needed and found additional items to purchase from browsing.

#### Theme 3: there are pros and cons with shopping instore and online

Most participants described how they use both online and physical stores to shop for their groceries. Most commonly, participants discussed how they preferred to shop instore for delicate or perishable items – especially meats, fruits and vegetables – because online they were not able to physically pick up items to inspect their quality:

*I don’t like buying my [fruit and vegetables] online because I really like to see and choose*. **(55-year-old female, low income)**

These perceptions did not vary across socio-economic groups. Other participants found that the convenience offered through online grocery shopping mitigated occasional experiences with poor-quality fruit and vegetables and that they understood that sometimes supermarket staff may accidentally choose less fresh items for online orders due to their busy workload.

Almost all participants described how searching for healthy food online was more time-consuming than when shopping instore, and that this sometimes disincentivised online purchasing:

*I think the healthier options tend to get lost in the noise of online*. **(32-year-old male, mid-high income)**

Participants commented that the act of physically seeing an item instore was often a purchase trigger for them to remember to purchase a particular item, leading some to describe how they do not shop ‘well’ online due to forgetting things. Seeing a photo of a product on the supermarkets’ websites was not viewed as an effective purchase trigger compared to physically seeing the item instore.

Similarly, participants believed that physically seeing fresh fruit and vegetables instore helped to put them in a ‘healthier mindset’ and influenced their other instore purchases. Participants also commented that the instore marketing techniques used for fruit and vegetables, such as store layout and vibrant colours, influenced them to buy more of them than they did online:

*I think the benefit from walking in the shop is you get to see the [fruits and vegetables]. If it does look [like] good, seasonal fruit… I’ll buy it if I see them and they look good… You can’t do that in online shopping. So you don’t have those impulse buys for fruit because you never really know what you’re looking at.* **(38-year-old female, mid-high income)**

Some participants suggested that the ‘physically detached’ aspect of online grocery shopping meant that they did not have to think too hard about food decisions which led them to choose less healthy options. On the other hand, some participants described how this ‘detached’ feeling meant that they purchased less unhealthy snack foods online because they did not experience cravings compared to when shopping instore:

*Instore, I will purchase different items because I can see the physicality of them*. **(37-year-old female, low income)**

Another benefit of shopping instore, compared to online, included the relative ease of reading nutritional information. This was particularly salient for label information related to allergens and country of origin. Some participants discussed that not being able to physically hold an item to read its nutritional information was a negative aspect of online shopping, especially if trying to compare the nutritional information of two similar products and make the ‘healthiest choice’. Participants described how it was hard to locate nutritional information online, and if they did locate it, the information was often incomplete or missing. Additionally, participants who used their mobile phones to do their online grocery shopping commented that the photos with nutritional information were often very small and hard to read. These perceptions did not differ across socio-economic groups.

Convenience was the most appealing aspect of online grocery shopping for most participants. Using online grocery platforms instead of shopping instore was generally reported to save parents and carers time and reduce stress because they did not need to bring their children with them to grocery stores. Taking children instore to shop was described as an unpleasant experience. Being able to place the online order at a time that suited them, such as after children were asleep, was seen as beneficial:

*You’ve got time to sit down in your comfort and gloss over the catalogues and what’s available and all the specials that come in and then put them all into your cart. So it’s just a lot more time that you can spend on doing your grocery lists then say, you know, if you were just in the supermarket you’re running around with the child and…. I mean you can carefully plan lot more things*. **(55-year-old female, low income)**

The majority of participants described how there was an event that triggered them to swap to online grocery shopping when they had previously shopped instore. These trigger events included the COVID-19 pandemic, having a baby and experiencing particularly busy periods at work, or having a health issue.

### Shoppers’ perceptions of how online grocery retail environments can be modified to better support healthier grocery purchases

#### Theme 4: scepticism of supermarket-led actions

Participants generally had a sense of distrust towards supermarkets. For example, participants reported being wary of ‘healthy’ product promotions or suggestions from online supermarkets, as they believed that supermarkets are not objective in their assessment of what is healthy and are influenced by their relationship with suppliers. Participants perceived that supermarkets were focused on making money and operating within their capacity as private businesses, being more concerned about making profits than they were about the healthiness of the products they sold. However, a few participants suggested that it was not the place of government to try to interfere with a supermarket’s ability to make money. Despite this scepticism towards supermarket-led change, some participants felt strongly that supermarkets could, and should, do more to make healthy food and beverages more affordable and accessible when shopping online.

Specific supermarket-led initiatives that were suggested included the use of a filter or specific ‘healthy’ food category, so it was easy to avoid seeing unhealthy items when grocery shopping online. Despite not engaging with current search optimisation techniques, this concept was suggested by some participants to ensure healthier items were presented higher in the search results list.

The online catalogue was also perceived as a potential avenue for promoting in-season fruits and vegetables, and participants believed it would be beneficial to offer price promotions on these kinds of items. Simple online messaging or advertisements from healthy food growers and producers were suggested as a possible way to counter the powerful messaging and promotions available online from unhealthy food brands and companies.

A few participants thought supermarkets could discourage unhealthy purchases by including a warning pop-up when an unhealthy food item was added to the online grocery basket, or by including a prominent online warning label on individual products that are high in unhealthy nutrients.

#### Theme 5: a role for government-led actions

Although the opinions of participants were mixed, they generally agreed that there was a role for the government to regulate online supermarkets’ ability to promote and market unhealthy food and beverages. However, there was some belief that this could be difficult due to resistance from supermarkets and unhealthy food and beverage manufacturers. Participants believed that regulations to restrict unhealthy food and beverage marketing through online supermarkets was in the national interest and cited concerns about how unhealthy foods and beverages were creating a ‘cost to society’ through medical conditions and healthcare costs. Participants compared unhealthy food to tobacco, especially in terms of regulation and marketing, with one participant indicating:

*Junk food is the cigarettes from 30 years ago.* **(50-year-old male, mid-high income)**

Participants also commented that government-led policies could ensure that healthy foods were more widely available online and could improve the transparency and objectivity about whether a food is actually healthy or not. Many participants suggested that a government-led general online healthy eating awareness or education campaign could be useful to help individuals choose healthier foods when they shop online for groceries. A government policy to reduce the cost of healthy food relative to unhealthy food was suggested as another way to improve the healthiness of online supermarkets.

Not all participants agreed that government regulation was necessary or appropriate, with a few participants reporting that it is a person’s own responsibility to eat healthily, and that individuals should be allowed to make their own decisions regarding what they choose to buy and eat. Participants on lower incomes particularly discussed how people cannot be ‘forced’ into choosing healthy food and questioned whether there was anything that the government could do to improve population diets and therefore the healthiness of online supermarkets:

*Some people don’t want to be healthier, unfortunately, it’s their lifestyle choice… so I honestly I don’t know what the government could do because you can’t really force stuff on people*. **(42-year-old female, mid-high income)**

## Discussion

This is the first study to explore Australian shoppers’ perceptions of online grocery shopping and the potential influence of this on the healthiness of purchasing behaviours. We found that the parents and carers who used online platforms for grocery shopping did so primarily for convenience. Participants perceived that unhealthy foods were more likely to be price-discounted and advertised on the supermarkets’ webpage while shopping, compared to healthier options, which made it hard for them to purchase healthy groceries. Participants were generally sceptical about the motives and therefore impact of supermarket-led actions to improve the healthiness of online grocery retail platforms, but most believed that both supermarkets and governments have a role in making online grocery platforms more supportive of healthy food and drink purchases.

Most participants, particularly those with lower household income, viewed the extensive prevalence of online grocery price promotions as favourable for shopping on a budget. For online grocery platforms, these price promotions were described as being accessible not only at the point of sale for a given product but also in the online catalogues where products could be added to a shopper’s cart seamlessly. These online price promotions were generally perceived to be more common for unhealthy, compared to healthy, foods. This perception is in line with the literature, which shows that in Australia, unhealthy foods and drinks are discounted to a greater extent and magnitude compared to healthy items, making them more appealing to those on a lower income^(11,30)^. Policies to restrict price promotions on unhealthy food and drinks across both instore and online grocery platforms have been suggested by public health groups as a means of improving population diets and overall health^(31)^. In the UK, a ban on volume-based price promotions (i.e. multi buys or ‘2 for 1’) in both instore and online grocery settings has been enacted into law; however, implementation is yet to take place^(32)^.

Another concern raised by participants in our study was the inadequate nutrition labelling for products sold online, with this information often being too small or illegible for reading. These findings support previous studies that have described the availability of nutritional information online as being inconsistently presented^(33–35)^. Specifically, a 2021 Canadian study by Lee *et al.* found that although all products examined by the researchers had photos of their nutritional information available, this information was presented in the form of photographs that were illegible 88 % of the time^(34)^. Similarly, a study of three major UK online supermarkets by Moore and Wallis found that front-of-pack nutrition labelling was displayed inconsistently, with labelling present in photos of 52 % of sampled products^(35)^. A UK study by Stones found that although nutritional information was provided through online grocery retail platforms, to locate this information required shoppers to click into a separate window to bring up a product’s description^(33)^. In many countries, labelling laws mandate nutritional information on all foods and beverages, with specifications for size and legibility. These requirements should translate to online grocery retail platforms.

Our finding of participants’ dissatisfaction with shopping online for fruits and vegetables, and other perishable items, due to quality concerns, and a distrust of supermarket staff to select high-quality items is also in accordance with the literature. For example, a US study interviewing low-income recipients of the Supplemental Nutrition Assistance Program (SNAP) found that participants felt that shopping online could remove their autonomy over choosing which fresh and perishable items they bought and that they disliked this feeling of reduced control^(36)^. Similar concerns were also raised by participants receiving SNAP in a study by Rogus *et al.*, with doubts that the fruit, vegetables and meat selected by supermarket staff for online orders would not be as fresh as if participants had selected it themselves^(37)^. A 2022 study of SNAP-eligible households in the USA found that households that shopped online for groceries reported purchasing less fresh produce (OR = 0·34, *P* < 0·001), meat and seafood (OR = 0·29, *P* < 0·001) and sweets (OR = 0·54, *P* = 0·005) than those households that shopped instore^(38)^. Methods or strategies to encourage purchasing of fruit and vegetables online is an area that could be investigated in further research.

Participants in our study discussed how a key benefit of online grocery shopping is being able to have a greater degree of control over their spending, thereby finding it easier to adhere to a grocery budget because of the visibility of a running total as an online shopping cart is updated. These online functions to support budget-conscious shoppers is highly salient today, as the cost of living increases across the world. Globally, prices on grocery items are trending upwards due to inflation, climate change, ongoing supply chain issues following the COVID-19 pandemic and the War in Ukraine, amongst other things^(39)^. These increased costs of living mean that many Australian households are trying to reduce their grocery budget^(40)^. Online grocery shopping may become more attractive to those who are keeping tight budgets.

Our findings were mixed with regard to how easily online grocery shopping makes healthy purchases, compared to instore. This is in contrast with the prior literature, which generally describes shopping online as more health-promoting than shopping instore. For example, some studies have described online grocery retail platforms as more health-promoting than instore equivalents as shoppers are not exposed to persuasive instore marketing tactics^(41,42)^. Similarly, a 2019 pilot trial to alleviate food access disparities among twenty individuals living in transport-scarce and low-resource areas found that those who were randomly assigned to a 1-month use of online grocery delivery services purchased a greater proportion ‘green’ (healthiest) foods and a lower proportion of ‘red’ (least healthy) foods, compared to the control group who shopped instore (54 % *v*. 25 %, and 22 % *v*. 46 %, respectively, p)^(42)^. Similarly, a 2017 study by Huyghe et al found that shoppers spent less on ‘vice’ items (chocolate, chips, salty snacks, etc.) when shopping online compared to instore^(41)^. A mixed methods study with SNAP-eligible households reported that online grocery shoppers perceived they purchased fewer impulse items, like sweets, when shopping online^(38)^. In reality, both instore and online grocery retail environments promote highly processed unhealthy foods^(43)^ and encourage unhealthy food purchases. It is not surprising that participants in our study reported that the marketing of unhealthy foods in both environments (online and instore) leaves shoppers feeling ‘saturated’ with messages encouraging the consumption of unhealthy food.

The use of online grocery platforms is likely to continue to grow. The COVID-19 pandemic was described by many participants as a trigger to begin shopping online for groceries. The onset of the pandemic changed the global methods of shopping for essential grocery items due to lockdowns and fears that shopping instore in crowded supermarkets may lead to exposure to the virus^(44)^. Australia saw particularly large shifts in the manner of purchasing, with online sales for food rising from AUD$521 million to AUD$896 million between March 2020 and June 2021^(45)^, with online sales continuing to rise to AUD$1112 million in May 2023^(13)^. Internationally, online grocery sales rose by 13·7 % year on year in the USA^(46)^ and increased by £13·5 billion between 2019 and 2022 in the UK^(47)^. Despite now living without COVID-era lockdowns or restrictions, online grocery retail platforms are still increasing in popularity. In 2023, the use of online grocery platforms in Australia is projected to generate US$8·06 billion in market revenue, an increase from US$6·33 billion in 2022^(48)^. This highlights the need to ensure online grocery retail platforms promote healthy food purchasing.

Participants had a range of novel ideas for improving the healthiness of online grocery platforms. A few participants commented that creating a ‘healthy’ category could prompt them to choose healthier products online. A previous study focusing on a narrow category of healthiness found that a healthy option prompt in an experimental online supermarket increased purchasing of fibre across cereals, bread and crackers^(49)^. Conversely, another study using an experimental supermarket found that healthy option prompts did not decrease total purchase energy density because few prompted products were selected by participants^(50)^. Previous literature has shown that the high visibility of unhealthy food and drinks in supermarkets influences purchasing^(51)^; therefore, making these products less visible online could lead to healthier shopping. However, in general, participants were sceptical about how much supermarkets themselves would do to make their online shopping environments healthier as they are ultimately driven by profits. On the other hand, most participants were generally in favour of government-led policies such as restrictions on unhealthy food and beverage price and placement promotions through online grocery stores, and education or awareness campaigns for general healthy eating specially tailored to online supermarkets. Whilst the latter is important, it should be only considered alongside a complementary suite of other actions to improve the online grocery food retail environment.

### Strengths and limitations

Strengths of this study include the novelty of the research topic, as this is the first study to our knowledge to explore the perspectives of Australian online grocery shoppers in this way. Additionally, the reflexivity of the research team was acknowledged throughout the data collection and analysis, thereby minimising potential bias in our results. Using a theory such as the Marketing Mix framework to underpin the development of the interview guide is another strength as it enabled a rich discussion on various a priori-defined marketing techniques, which, despite being developed many years ago, are still relevant for online grocery shopping.

The limitations of this study firstly relate to using a paid recruitment company to recruit participants, as this can mean that those who participate are not truly representative of the ‘average’ online grocery shopper and may instead be those who are more interested in research generally. However, we attempted to minimise such bias by ensuring that the recruitment company used was reputable, as they sampled participants from a variety of locations across Australia and minimised oversampling of the same participants by excluding participants from participating in more than one interview in 6 months. Also, interviews were primarily coded by one author, which could introduce bias in the interpretation of data. However, this was minimised by collaboratively coding one interview with two co-authors to gain a greater depth of interpretation before one author coded the remaining interviews.

As this study was qualitative in nature, the findings cannot be generalised to all Australian parents who shop for online groceries. Another limitation was that this study only focused on families with school-aged children. Some participants described how they initially started online grocery shopping after having a baby, so by excluding those whose children are too young to attend school we may be missing a key demographic of online grocery users. Future research should include more diverse groups, including those from varied socio-economic groups and contexts, and confirmed with additional quantitative and qualitative studies.

### Conclusion

While experiences with online grocery shopping varied, overall participants preferred shopping online for their groceries. They expressed that it is convenient and allows them to better stick to their planned budget. However, it was expressed that online environments are saturated with unhealthy food promotions, and purchasing healthy foods could be difficult. Efforts to improve population diets should consider how novel policies can be adopted and enforced into online food retail platforms, and more research is required to understand what new policies are required to address the unique aspects of the online shopping experience to better promote healthy food selection.

## Supporting information

Bennett et al. supplementary material 1 Bennett et al. supplementary material

Bennett et al. supplementary material 2 Bennett et al. supplementary material

## Supplementary material

For supplementary material accompanying this paper visit https://doi.org/10.1017/S1368980024001046
