# Supplementary material for: ‘Healthier options tend to get lost in the noise of online’ – Australian shoppers’ experiences with online grocery platforms
Source: Public Health Nutr. 2024 May 14;27(1):e134. doi: 10.1017/S1368980024001046 (PMC11148825; doi:10.1017/S1368980024001046)
Supplement: Bennett et al. supplementary material 1 — Bennett et al. supplementary material [file S1368980024001046sup001.docx]

## Appendix A:

Example of questions asked, guided by the Marketing Mix Theory framework:

| Marketing mix theory framework component | Example questions |
| --- | --- |
| Introductory questions | - Why did you start shopping for groceries online? - Who do you shop for when you purchase your groceries online? (In the context of family members) |
| Place (online grocery retail) | - When you shop online for your groceries, why do you choose an online platform rather than in-store? *Prompt: what are the benefits?* - How often do you shop online? *How does this compare to instore?* |
| Product (groceries) | - Do you use a shopping list to plan in advance what you are going to buy online? *To what extent do you stick to that? For unplanned things, what else influences you on what to buy?* - Do you think the foods you buy online for a given shop would be similar if you had shopped in-store instead? |
| Price (value) | - Do you think you spend more or less when shopping online? Why do you think this is? - How much do you spend in an average month using online grocery sites? Is this different to in-store spending? |
| Promotion (advertising, social media, price promotions) | - When shopping for groceries online, are you aware of “specials” or price promotions? *Can you describe these and how you use them?* - What types of foods are usually price promoted when you’re shopping online? - Have you noticed prompts for “healthier choices” while you shop online? *What do you think about them?* |
| What should be done to make online grocery retail healthier? | - The World Health Organization recommends that supermarkets stop promoting junk food at cheap prices and instead promote healthier options. What do you think about this recommendation? - Are there other things that online food retailers could do to support you and others to purchase healthy foods? - Who do you think is responsible for healthy online food retail environments? Do you think governments have a role to play by regulating online supermarket practices? - What else do you think supermarkets and governments can do to make it easier for you to purchase healthy foods? |
