# Supplementary material for: ‘Healthier options tend to get lost in the noise of online’ – Australian shoppers’ experiences with online grocery platforms
Source: Public Health Nutr. 2024 May 14;27(1):e134. doi: 10.1017/S1368980024001046 (PMC11148825; doi:10.1017/S1368980024001046)
Supplement: Bennett et al. supplementary material 2 — Bennett et al. supplementary material [file S1368980024001046sup002.docx]

| **THEME** | **MAIN CODE** | **CODES** |
| --- | --- | --- |
| ***Online promotions are useful, but unhealthy food specials dominate*** | **Unhealthy food marketing online is too prominent** | Harder to buy healthy food online compared to in store  Healthy food is not on special often  If healthy food isn't as visible to you, you won't buy it  Not enough price promotions on healthy food  Presence of price promotion influences purchasing of unhealthy food  Promotions on supermarket home page  Prompt on website to browse through specials  Should promote both healthy and unhealthy foods  Specials are not common for fruit or veg  Unhealthy food promotions are too prominent |
|  | **Impulse buying online** | Browsing behaviour online  Impulse buying because of price promotion  Impulse buying unhealthy food in store  Impulse buying unhealthy food online  In store allows for browsing for specials more than online  Junk food specials more obvious when shopping in store  Junk food specials more obvious when shopping online  More browsing online than in store  More impulse buys online  More impulse buys instore  Price promotions can trigger impulse buying |
|  | **Experiences with personalised marketing online** | Feels misled by promoted search results  Has noticed promoted search results  Hasn't noticed promoted items in search results  Ignores promoted search results  Has seen healthy option prompt  Hasn't seen healthy choice prompt before  Have you forgotten prompt is mostly for unhealthy food  Personalised specials list  Unhealthy items promoted in search results  “Have you forgotten?” prompt |
|  | **Using loyalty points or reward programs online** | Buying extra items for loyalty points  Spending more to qualify for bonus loyalty points  Loyalty points online |
|  | **Opinions on online recipes** | Feature to add directly from recipe page to shopping cart  Has seen recipe prompt but not tried  Has used recipes before  Has not seen or used recipes online  Meal planning using recipes  Positive response to recipe prompt  Recipe prompt not relevant due to different dietary preferences  Budget recipes ideas are useful to save money |
|  | **The importance of the online catalogue and price promotions** | Begin shop with reading online catalogue  Begin shop with looking at specials tab  Buying extra to take advantage of the price promotion  Don't use catalogue online  Easier to stay within budget using specials when shopping online  Participant with low income - importance of catalogue  Online catalogue encourages impulse buys of price promotion  Online only specials  Positive feelings about price promotions  Shop directly from online catalogue into cart  Shopper actively seeks out price promotions online  Using the online catalogue  Positive feelings about price promotions |
| ***Online grocery platforms are helpful for budget conscious shoppers*** | **Pricing tactics** | Spend less money shopping online  Spend less money shopping instore |
|  | **Affordability** | Cost of food rising  Sort by unit price  Healthy food is more expensive than unhealthy food  Price has a big influence on purchase decisions  Reducing the price of healthy food would have large impact on purchasing habits  Try to save money by buying things on price promotion  Online budget recipes  Not able to access reduced to clear items online  Not everyone can afford a lot of fresh food  Variability in fresh produce prices makes them hard to budget for |
|  | **Delivery fees** | Free delivery enticed beginning to online shop  Paying a subscription fee for unlimited deliveries  Spending a specific amount to get free delivery  Delivery costs are too expensive |
|  | **Keeping to your budget when shopping online** | Able to check basket and purchase price before checking out  Easier to keep track of spending online  Unexpected total spend when in store  I can stick to my list online |
|  | **Pre-planning purchases when shopping online** | Begin shop by looking through “bought before” list  Using a category to search for items  Before online shopping has a clear idea of what to purchase  Checking inventory at home before doing online shopping order  Doesn't plan shopping with a list  Online shopping reduces food waste  Sticks to the shopping list online |
| ***There are pros and cons with shopping instore and online*** | **Hybrid shopping – both in store and online** | Buy some items in store and some items online  Shopping in store for delicate or perishable items |
|  | **Reading nutritional information online** | Are photos of ingredients lists current online?  Check nutritional information more in store than online  Comparing nutritional info is hard online across two products  Physically comparing two products in store  Doesn't compare nutritional information across two products online  Doesn't read nutritional information for every product  Reading country of origin information is important  Understanding nutritional information is difficult  Using a phone to online grocery shop means that nutritional information photos are very small and hard to read |
|  | **Issues with the quality of fruit and vegetables purchased online** | Buy minimal fruit and veg online, only necessities  Buying fruit and veg in store instead of online  Do supermarkets give less fresh items to online orders?  Doesn't buy fruit and veg online  In store marketing for fruit and veg is effective  No quality concerns with fruit or veg bought online  Wants to personally select fresh items in store |
|  | **Feelings and emotions influence purchasing behaviours** | Emotions can influence purchasing of unhealthy food  Feelings or emotions influencing purchasing (general)  More susceptible to cravings when shop in store  Online shopping is relaxing, a pass time  Don’t have to think too hard when shopping online |
|  | **Differences in the shopping experiences because you can’t physically see products** | Difficulties because can't physically see product  Forget things when shopping online because not prompted by physically seeing items  Physically seeing fresh fruit and veg puts you in a healthier frame of mind for purchasing food  Seeing a photo online doesn't influence purchasing as much as seeing the physical product in store  Hard to make the healthiest choice when you can’t physically see items  Hard to visualise how much food you are buying |
|  | **Convenience** | Able to select delivery for a time that suits you  Don't have to physically walk down aisles in supermarket  Health condition means spending a long time shopping in store is difficult  Less time consuming to shop online compared to instore  Online grocery shopping is easier when you don't have a car  Online shopping is more convenient  Online shopping is relaxing, a pass time  Online shopping means you don't have to bring children to the supermarket  Pandemic made us shop online  Use online shop because it's faster than in store  You are able to online shop in your own time, when it suits you  Online shopping is faster than in store  There are pros and cons to shopping online |
|  | **Children influence how the family shops and what they buy** | Children and teenagers are susceptible to food marketing practices in-store  Children have selective taste preferences and may not want what's healthy  Concerned about child’s nutrition  Trying to be good eating role models for children  Taking children in store to shop (negative) |
|  | **There is no difference in the accessibility of healthy food online or instore** | Healthy food not more or less accessible online |
| ***Scepticism of supermarket-led actions*** | **Supermarkets operate as private entities** | Don't need government intervention into supermarket regulation  Government shouldn't interfere with private companies  Online shopping is changing the supermarkets business model  Supermarkets are responsible for regulating themselves |
|  | **Distrust of supermarket and scepticism of their motives surrounding health** | Scepticism of health star ratings  Scepticism of nutritional information provided by supermarkets  Supermarket regulation won't happen without government intervention  Supermarkets are trying to make money  Supermarkets sell a lot of unhealthy food  Supermarkets should do more to promote health  Supermarkets should do more to put healthy food on price promotion  Supermarkets think more about money than about healthiness of products  Health information from supermarkets isn’t objective  Price promotions are for items that are poor quality |
|  | **Suggestions for policies to improve the healthiness of online grocery retail environments** | Supermarket initiative suggestion  Awareness or education campaign for healthy food  Filter by health star rating to improve online shopping for healthy foods  Filter option to remove unhealthy food from online  Have a healthy food online tab or category online  Healthcare spending could increase due to poor dietary choices  Healthy option prompt would help with purchasing decisions  Is it everyone's job to make supermarkets healthy?  It would be hard for the government to regulate supermarkets  Make healthy food specials more prominent online  Supermarkets should make it easier for people to find healthy food online |
| ***A role for government-led actions*** | **How to regulate online supermarkets** | Can't force people to buy only healthy food  Government policy to regulate supermarkets  Government should have a big role in regulating supermarkets  Government should look out for people's best interest  Government should regulate what supermarkets are allowed to promote and advertise  Taxes on unhealthy foods |
|  | **Personal responsibility narratives** | All food can fit in your diet, even unhealthy food  Comparing junk food to tobacco  Government overreach in food policy  Individual choices  Judgement of others for not knowing cooking basics  Personal responsibility  Personal taste preferences override health recommendations |
|  | **Role for government** | Positive response to government regulation of online supermarkets, but acknowledge that unlikely to happen  Positive response to WHO guidelines about marketing unhealthy food online  Unequal power of fresh food vs unhealthy food companies  Unsure whose job it is to make supermarkets healthier  Policy suggestion |
